# Skin Type Diversity in Skin Lesion Datasets: A Review

**DOI:** 10.1007/s13671-024-00440-0

**Published:** 2024-08-14

**Authors:** Neda Alipour, Ted Burke, Jane Courtney

**Affiliations:** School of Electrical and Electronic Engineering Technological, TU Dublin, City Campus, Dublin, Ireland

**Keywords:** Fitzpatrick skin type · Skin lesion datasets · Skin type diversity · Deep learning

## Abstract

**Purpose of review:**

Skin type diversity in image datasets refers to the representation of various skin types. This diversity allows for the verification of comparable performance of a trained model across different skin types. A widespread problem in datasets involving human skin is the lack of verifiable diversity in skin types, making it difficult to evaluate whether the performance of the trained models generalizes across different skin types. For example, the diversity issues in skin lesion datasets, which are used to train deep learning-based models, often result in lower accuracy for darker skin types that are typically under-represented in these datasets. Under-representation in datasets results in lower performance in deep learning models for under-represented skin types.

**Recent findings:**

This issue has been discussed in previous works; however, the reporting of skin types, and inherent diversity, have not been fully assessed. Some works report skin types but do not attempt to assess the representation of each skin type in datasets. Others, focusing on skin lesions, identify the issue but do not measure skin type diversity in the datasets examined.

**Summary:**

Effort is needed to address these shortcomings and move towards facilitating verifiable diversity. Building on previous works in skin lesion datasets, this review explores the general issue of skin type diversity by investigating and evaluating skin lesion datasets specifically. The main contributions of this work are an evaluation of publicly available skin lesion datasets and their metadata to assess the frequency and completeness of reporting of skin type and an investigation into the diversity and representation of each skin type within these datasets.

**Supplementary Information:**

The online version contains material available at 10.1007/s13671-024-00440-0.

## Introduction

Diversity is an important feature in datasets used for training artificial intelligence (AI) based models, as the performance of AI is only as good as its data. In this paper, “skin type diversity” refers to the range and representation of different skin types within the human skin image dataset. It provides the opportunity to verify the comparable performance of a trained model for each skin type. Authors frequently report ethnicity instead of skin type, but ethnicity and skin type are not the same, as many ethnicities can have diverse skin types. There are some other works whose datasets focus on the representation of varied ethnicities [1]. Ethnicity is a wider and more complex concept that refers to groups characterized by shared geographical, ancestral origin, cultural, religious, linguistic, or other shared characteristics [2]. Sufficient diversity should encompass a range of skin tones that adequately represent the population being studied, enabling an assessment of whether a particular skin type is under-represented to a degree that it impacts the reliability of the AI model [3]. Addressing this issue is crucial, as it can result in AI models favoring the majority class, reducing accuracy for the minority class. Techniques like resampling, cost-sensitive learning, over-sampling, under-sampling, and ensemble methods help to balance datasets and improve models’ performance [4, 5]. These approaches are particularly useful for addressing insufficient skin type diversity by ensuring that all skin types are adequately represented and learned by the model, thereby enhancing the model's ability to perform comparably well across different skin types.

In examining the performance of AI on human skin, particularly regarding its lower accuracy for dark-skinned individuals, it is important to recognize that the observed disparities may not solely be due to algorithmic bias. It might be attributed to broader systemic inequities in data collection, demographic characteristics of participants, their socioeconomic status, and other sociological factors [6, 7]. In this paper, the term bias refers to the inadequate representation of skin types in the training datasets and the resulting difference in the performance of trained AI models for certain skin types [8]. This issue can potentially lead to the exclusion of certain groups of people by AI-based models.

The effect of inadequate skin type diversity and under-representation of dark-skinned people in datasets can be seen in many AI-based technologies. For example, AI systems that judge beauty pageant winners are biased against darker-skinned contestants [9]. In a beauty contest run by Beauty.ai, the 44 finalists were judged by the algorithms as the most attractive, except for six who were described as “Asian”, and all were described as “white”. Only one finalist was dark-skinned [10, 11]. Another study investigating the performance of object detection systems on pedestrians with different skin types showed higher precision on lighter skin types than on darker skin types [12]. In another work, bias in face verification applications and datasets was evaluated concerning different skin types, and found that recognition accuracy was reduced for darker-skinned people [13]. The effect of this issue on the performance of robotic systems such as a robot peacekeeper, a self-driving car, and a medical robot was assessed [14]. It was shown that current AI and robotic systems have lower performance for certain skin types.

In AI in healthcare sectors, there are consumer wearable devices that are used for tracking activity, sleep, and other health-related purposes, but due to some limitations, these health products may only be useful for light-skinned people. Findings show that these devices are inaccurate, and even may not work at all for dark-skinned people [1, 15]. While other literature has pointed out potential inaccuracies in pulse oximetry for individuals with darker skin tones, the findings show that the Apple Watch, which employed the Fitzpatrick skin type scale in its model, did not exhibit such limitations seen in traditional pulse oximeters that can be affected by skin pigmentation and performed consistently across different skin types [16]. However, the mentioned examples indicate that the needs of darker skin population groups are not well-represented [17], which can potentially lead to reduced accuracy for dark-skinned groups by deep learning-based models. Several factors play a role in the biased performance of these models towards dark-skinned people.

A significant reason among these is the lack of skin type diversity in datasets used for training AI-based models, the absence of reliable labels for each sample, and consequently, a lack of evaluation of the model's performance on a per skin type basis [1, 15]. There are many reasons for not having enough data from dark-skinned people in datasets used for AI applications. For example, in the case of skin lesion datasets, reasons include low incidence of skin cancer in dark-skinned people [18, 19], unequal access to healthcare [20], poor quality images due to poor quality of care [21, 22] and algorithms with different performance for certain groups of people used in digital cameras as well as computer software [23, 24] contribute to unbalanced datasets. Consequently, dark-skinned people are under-represented in datasets from health services as well as research datasets [20]. Deep learning-based models trained on lighter-skinned subjects are at risk of poor performance for people with darker skin [25].

**Fig. 1:**
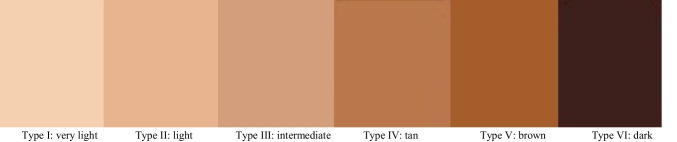
The range of skin tones in the Fitzpatrick skin type scale classifies skin tones into six types

Due to the problems mentioned above, it is necessary to evaluate and quantify skin type diversity to detect under-representation in datasets before using them for training AI systems. Doing this helps to prevent models from having a lower performance for darker-skinned groups of people [26]. The Fitzpatrick scale might be helpful in this regard which provides a skin tone classification based on reaction to exposure to sunlight [27]. While it provides a useful framework for categorizing skin types, it may not fully capture the full spectrum of the human skin diversity needed for training AI models [26]. This scale is used in dermatology to classify skin tones into six numbered categories as shown in Fig. 1. Despite its limitations, the Fitzpatrick skin type scale has previously been used to evaluate skin type diversity in datasets [26].

Although the issue of inadequate skin type diversity has been discussed in previous works, these have not attempted to evaluate skin type diversity for datasets. For instance, in the Gender Shades study [28], the Fitzpatrick scale was used to evaluate the PPB, IJB-A, and Adience datasets. However, rather than measuring skin type diversity over six separate Fitzpatrick skin type categories, the authors instead classify the images in these datasets using two aggregate groups—darker and lighter.

To mitigate discrimination against certain groups of people, the FairFace dataset was created, a balanced face image dataset for seven race groups that provides more accurate and consistent modeling across different race and gender groups [29]. However, this work focuses on ethnic diversity and does not report skin type diversity. A new method was proposed using computer simulations to detect biases in face detection using Bayesian parameter search in high dimensional feature space. Although the Fitzpatrick scale was considered for the identification of demographic biases in commercial face application programming interfaces (APIs), skin type diversity was not measured [30].

A new method was introduced for human skin detection, not using color information, but rather using a U-Net-based segmentation network [31]. This method was tested on two datasets containing face images: ECU (Edith Cowan University) and RFW (Racial Faces in the Wild). ECU is an imbalanced dataset created based on six different Fitzpatrick skin types and RFW is a balanced dataset with only the annotation of ethnicity, based on four test subsets: “Caucasian”, “Asian”, “Indian”, and “African”. In the case of the RFW dataset, it is not evaluated based on Fitzpatrick skin type but just based on ethnicity.

Casual Conversations was created, which is a fair and diverse dataset of videos collected from seven countries for AI applications, labeled based on the two skin tone scales of Monk [32] and Fitzpatrick [27]. Nonetheless, the authors do not report any measurement of skin type diversity for their dataset [33]. The SkinCon dataset was created for training models related to skin diseases, which contains labels for different skin types [34]. This dataset was constructed from two skin disease image datasets: Fitzpatrick 17 k [26] and Diverse Dermatology Images (DDI) [35]. Although the Fitzpatrick skin type scale is mentioned in this work, no measurement of skin type diversity is presented. Skin lesion image datasets were assessed for diversity based on their metadata including age, gender, ethnicity, and skin type. The authors mentioned that there is limited reporting on skin type in the metadata and also less representation of darker-skinned people in skin lesion datasets. However, the authors did not measure skin type diversity in any of the skin lesion datasets [1].

**Fig. 2:**
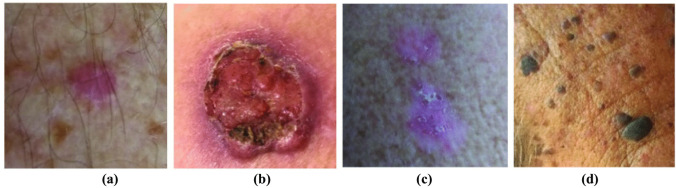
Some sample images from the PAD-UFES-20 dataset. (**a**) Skin type I. (**b**) Skin type II. (**c**) Skin type III. (**d**) Skin type IV

**Fig. 3:**
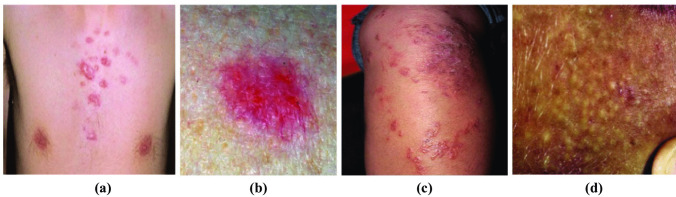
Some sample images from the Fitzpatrick 17k dataset. (**a**) Skin type I. (**b**) Skin type II. (**c**) Skin type III. (**d**) Skin type IV

To measure skin type diversity and detect under-representation in datasets used for training deep learning-based models, Fitzpatrick skin type metadata should be included in the datasets [26]. Accessing this information is a crucial step to not only detect under-representation in datasets, but also help to avoid training models on datasets with inadequate skin type diversity, and as a result prevent models from performing poorly for darker-skinned groups of people. According to our investigation, three available skin lesion datasets provide Fitzpatrick scale skin type metadata, labeled by dermatologists: PAD-UFES-20 [36], Fitzpatrick 17k [26], and DDI [35]. To investigate the issue of inadequate skin type diversity in datasets used for training deep learning models, just two datasets—PAD-UFES-20 and Fitzpatrick 17 k—are utilized as examples in this review. DDI was not used because it is a balanced dataset (albeit for three aggregate skin type groups, rather than for all six Fitzpatrick skin types). Sample images from the PAD-UFES-20 and Fitzpatrick 17k datasets are shown in Fig. 2 and Fig. 3, respectively.

Investigation of metadata in these two datasets is helpful to assess skin type diversity and check to what extent the lack of diversity in the datasets potentially leads to discrimination by models trained on the datasets. The main contributions of this study are an investigation into reporting skin type information in available skin lesions datasets, a significant extension of the work by [1], and an investigation into the diversity and representation of specific skin types within these datasets. Previous similar work by [37] discussed the lack of transparency in medical skin datasets and the necessity of demographic descriptions such as ethnicity and Fitzpatrick skin type for further analysis and deep learning applications. However, the authors do not address the potential limitations in skin type diversity within the investigated datasets, although the Fitzpatrick scale is included. Also, the two publicly available datasets, PAD-UFES-20 and Fitzpatrick 17 k, were published without thoroughly assessing skin type diversity by evaluating the representation of various skin types and ethnicities and ensuring a balanced distribution across different skin tones.

Given the examples of AI model underperformance for individuals with certain skin colors, having a reliable skin type label for each sample can significantly help address the under-representation issue in human skin-related databases, although alternative methods like skin type classification algorithms as a pre-processing step are also viable options [38]. Therefore, this study makes a significant contribution in this regard by:

1. Providing an investigation into publicly available skin lesion datasets to determine the extent of coverage in terms of reporting on skin types compared to other reported metadata in these datasets.

2. Presenting a comprehensive evaluation of skin type diversity level in three datasets where skin type metadata is provided. The results of this analysis are noteworthy, showing an inadequate representation of skin types in these datasets, which can be addressed by technical solutions.

This review emphasizes the danger of implementing algorithms on datasets lacking transparency and diversity, as supported by prior studies [39–41].

## Methods

The selection process used in our review to identify papers that used publicly available skin lesion datasets was based on the PRISMA statement [42]. The databases of PubMed, Elsevier, Springer, Google Scholar, and IEEE Xplore were searched. In our initial search, the following search terms were used: “skin cancer detection”, “skin lesion segmentation”, “skin lesion augmentation”, “balancing skin lesion datasets”, “skin lesion datasets”, “Fitzpatrick skin type metadata skin lesion”, “Fitzpatrick skin typology angle”, and “skin type diversity in skin lesion datasets” to identify papers on skin type diversity that make use of skin lesion image datasets. Table 1 provides a summary of which datasets are used in each of the selected papers. Section 3 includes a review of a subset of the identified datasets that match the following criteria: gender, age, ethnicity, and skin type.

**Table 1:** A subset of papers identified through the PRISMA process that used publicly available skin lesion datasets. We have attempted to select a subset that spans the majority of the skin lesion datasets used in the full list of identified papers. (C: Clinical images, D: Dermoscopic images)

Author	**Year**	**Dataset**	**Image**
Mendonça et al [43]	2013	PH2	D
Saez et al [44]	2014	Interactive Atlas of Dermoscopy	D
Sun et al [45]	2016	SD-198	C
Liao et al [46]	2016	AtlasDerm / Danderm / DermIS / Dermnet / Derma / DermQuest (Derm101)	D
Kawahara et al [47]	2016	Dermofit Image Library	D
Ge et al [48]	2017	MoleMap / ISBI-2016	D
Lopez et al [49]	2017	Dermofit Image Library / Dermnet / ISBI 2016 Challenge	D
Kawahara et al [50]	2018	7-point checklist	C
Han et al [51]	2018	Asan Dataset / MED-NODE	C
Gutman et al. [52]	2018	ISIC-MSK-2	D
Han et al [53]	2018	Edinburgh Dermofit Image Library / Hallym	C/ D
Shoieb and Youssef [54]	2018	DermQuest / MED-NODE / DermIS	C/ D
Goyal et al [55]	2018	ISBI 2017 / PH2 / HAM10000	D
Mendes and da Silva [56]	2018	MED-NODE / Atlas / Edinburgh	C/ D
Gonzalez-Diaz [57]	2018	2017 ISBI challenge / EDRA / ISIC Archive	D
Yang et al [58]	2019	SD-198 / SD-260	
Brinker TJ et al [59]	2019	MClass-D	C
Combalia et al [60]	2019	BCN20000	D
Xie et al [61]	2019	XiangyaDerm	C
He et al [62]	2019	Skin-10 / Skin-100	C
Pacheco et al [36]	2020	PAD-UFES-20	C
Han et al [63]	2020	SNU / Edinburgh	C
Han et al [63]	2020	Normal / Web	C
Milantev et al [64]	2020	SD-198 / MED-NODE / PH2 / SKINL2v2 / Seven-Point / Light Field Image	C/ D
Andrade et al [65]	2020	SMARTSKINS / Dermofit Image Library	C
Zhang et al [66]	2020	Skin-Cancer-Detection (SCD) / ISIC 2018	D
Hasan et al [67]	2021	Skin Cancer Benign vs. Malignant	D
Abhishek et al [68]	2021	Interactive Atlas of Dermoscopy / MClass-D	D
Maron et al [69]	2021	HAM10000 / PH2 / SKINL2 / BCN20000/ PROP	D
Krohling et al [70]	2021	PAD-UFES-20	C
Yao et al [71]	2021	ISIC 2018 / Seven-Point Criteria Evaluation (7-PT)	C/ D
Groh et al [26]	2021	Fitzpatrick 17 k	C
Abbas et al [72]	2021	Yonsei University Hospital	D
Ali et al [73]	2022	Monkeypox Skin Lesion Dataset (MSLD)	C
Alenezi et al [74]	2023	ISIC-2019, 2020	D

## Results

**Fig. 4:**
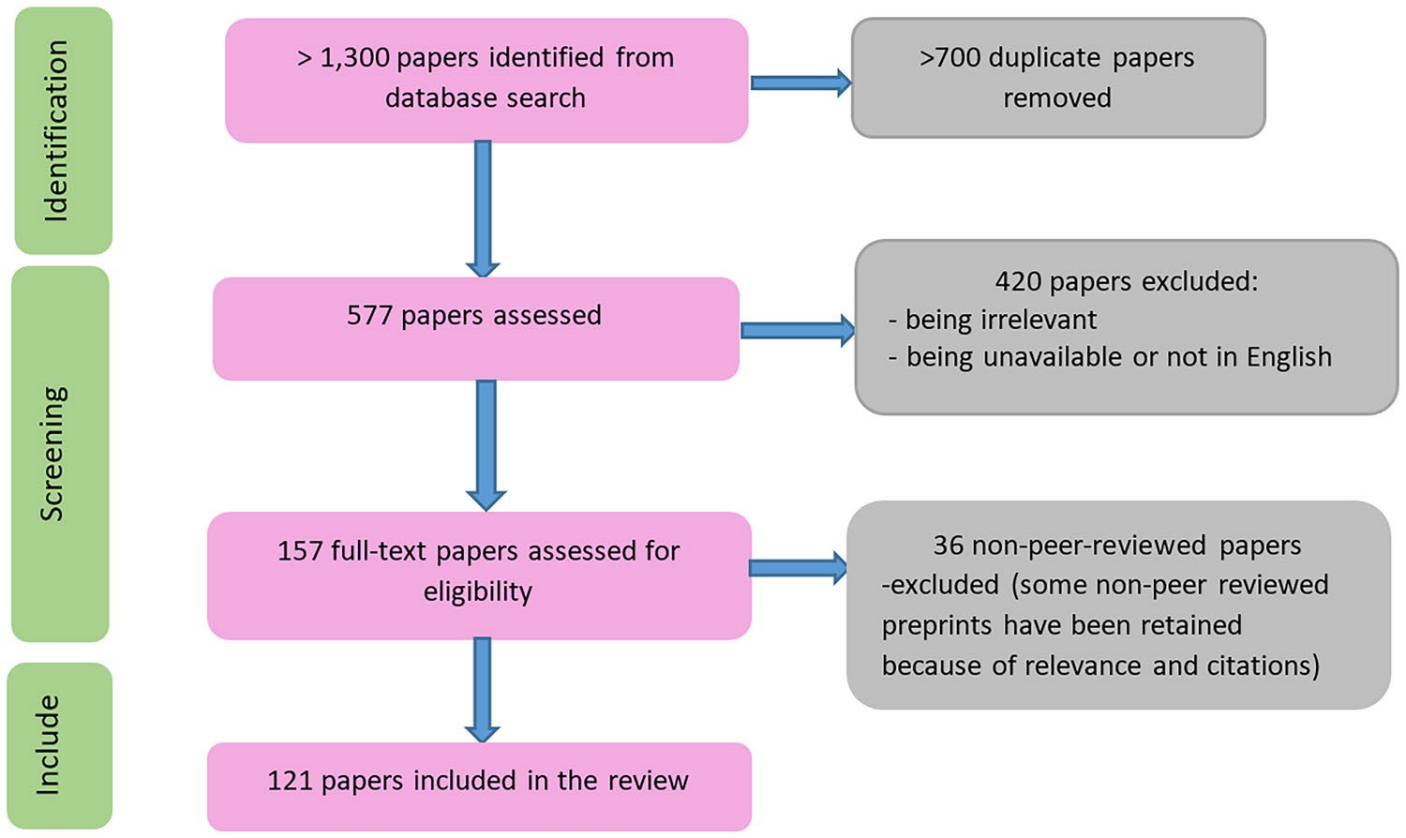
PRISMA flow chart of study selection

Our initial search (using the search terms listed in Sect. 2) returned over 1,400 publications as shown in Fig. 4. In the first screening, more than 800 duplicate papers were eliminated, leaving 690 papers to be assessed. In the second step, a further 513 publications were excluded due to lack of relevance (did not use skin lesion datasets), or being unavailable (including those not accessible without payment in Technological University Dublin), leaving 177 papers to be assessed for eligibility. Of these, 45 were excluded due to not being peer-reviewed. Ultimately, 132 publications were included in the systematic review.

The 132 papers identified from the search process used one or more publicly available skin lesion datasets. Table 1 shows a subset of these papers.^1^

As shown in Table 1, there are overlaps between papers using the same groups of skin lesion datasets. Through the process, 54 different skin lesion datasets were identified from these papers. Table 2 summarizes each dataset’s reporting of the following metadata: age, gender, ethnicity, and Fitzpatrick skin type. The number of images is also shown.

**Table 2:** 54 different publicly available skin lesion datasets used in publications and their reporting of four main metadata, showing a lack of reporting of skin type information to cover skin type diversity in datasets

Skin lesion datasets	No. Images	Metadata			
		Gender	Age	Ethnicity	Skin type
7-point criteria evaluation dataset [50]	> 2,000	✔	-	-	-
Asan [53]	120,780	✔	✔	✔	-
Atlas [56]	3,816	-	-	-	-
AtlasDerm [75]	9,503	-	-	✔	-
BCN20000 [60]	19,424	✔	✔	-	-
Cancer Genome Atlas [76]	2,860	-	-	-	-
Clinical Atlas [77]	839	-	-	-	-
DanDerm [78]	1,110	-	-	✔	-
Derm7pt [50]	> 2000	-	-	-	-
Derm101 [79]	107,656	-	-	✔	-
Dermatology Dataset [80]	336	-	✔	-	-
DermIS [81, 82]	7,172	-	✔	✔	-
Dermnet [46]	19,500	-	-	✔	-
DermNet NZ [75]	246	-	-	-	-
Dermofit Image Library [83]	1300	-	-	✔	-
Dermoscopic Atlas [77]	872	-	-	-	-
Dermoscopy Skin Lesion Multispectral Image Database [84]	30	-	-	-	-
DermQuest [81]	137	-	-	-	-
DDI [35]	656	✔	✔	-	✔
Edinburgh [85]	1,300	✔	✔	✔	-
EDRA Interactive Atlas of Dermoscopy [76]	1,000	-	-	-	-
Fitzpatrick 17k [26]	16,577	-	-	-	✔
Hallym [51]	152	✔	✔	✔	-
HAM10000 [77]	10,015	✔	✔	-	-
Interactive Atlas of Dermoscopy (IAD) [76]	> 2, 000	-	-	-	-
ISBI 2016 [52]	1,279	-	-	-	-
ISBI 2017 [86]	2,750	-	-	-	-
ISIC Challenge 2020 [87]	33,126	✔	✔	✔	-
ISIC-MSK [52]	225	✔	✔	-	-
ISIC-UDA [52]	557	-	-	-	-
Kaggle [75]	367	-	-	-	-
Light Field Image [88]	250	✔	✔	-	-
MClass [89]	100	-	-	-	-
MED-NODE [90]	170	-	-	-	-
MoleMap [82, 91]	102,451	-	-	-	-
Monkeypox Skin Lesion Dataset (MSLD) [73]	228	-	-	✔	-
Normal [63]	48,271	✔	✔	✔	-
OLE [46]	1,300	-	-	-	-
PAD-UFES-20 [36]	2,299	✔	✔	-	✔
PH2 [43]	200	-	-	-	-
SD-128 [45]	5,619	-	-	✔	-
SD-198 [45, 92]	6,584	✔	✔	✔	-
SD-260 [58]	20,600	✔	✔	✔	-
SIIM-ISIC Melanoma [87]	33,126	✔	✔	-	-
Skin-10 [62]	10,218	-	-	-	-
Skin-100 [62]	19,807	-	-	-	-
Skin Cancer’ Malignant vs. Benign [93, 94]	6,594	-	-	-	-
SkinCon [34]	3230	-	-	-	-
SkinL2 [88]	376	-	-	-	-
SMARTSKINS [95]	-	✔	✔	-	-
SNU [63]	2,201	✔	✔	✔	-
Web [63]	51,459	✔	✔	✔	-
XiangyaDerm [61]	107,565	-	-	-	-
Yonsei University Health System South Korea [96]	724	-	-	-	-

**Fig. 5:**
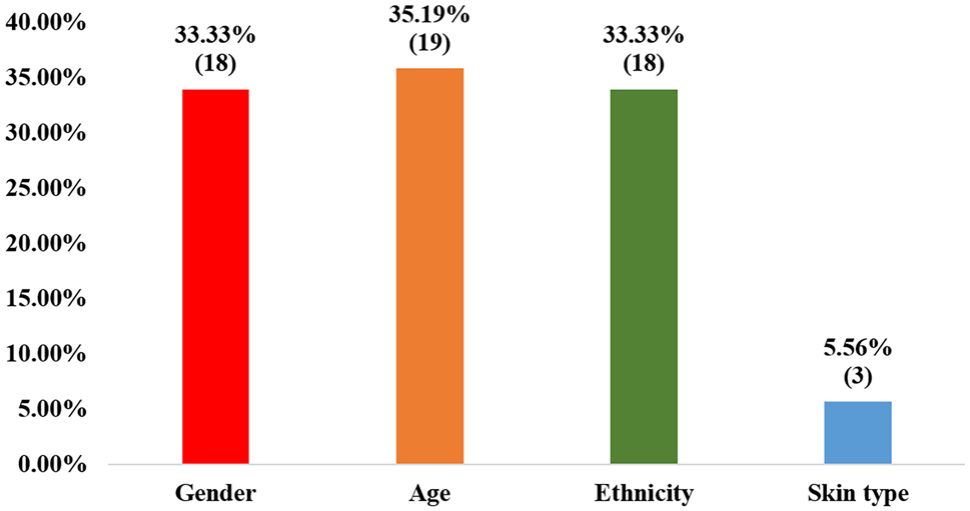
Percentage of the 54 skin lesion datasets that provide metadata for gender, age, ethnicity, and skin type respectively

Ideally, skin lesion datasets should achieve skin type diversity as well as have transparency in their metadata. As a result, not only would their diversity be easily measured, but also any imbalance would be detected before training models using these datasets. As seen in Table 2, only three datasets: PAD-UFES-20, Fitzpatrick 17k, and DDI provide metadata on skin type. They have skin type labels based on the Fitzpatrick rating system [1]. Figure 5 also shows the breakdown of reporting in the metadata for gender, age, ethnicity, and skin type. As shown, skin type metadata is the least frequently provided, being included in just 3 of 54 datasets (5.56%). Age metadata were the most frequently provided, being included in 35.19% of the datasets.

**Fig. 6:**
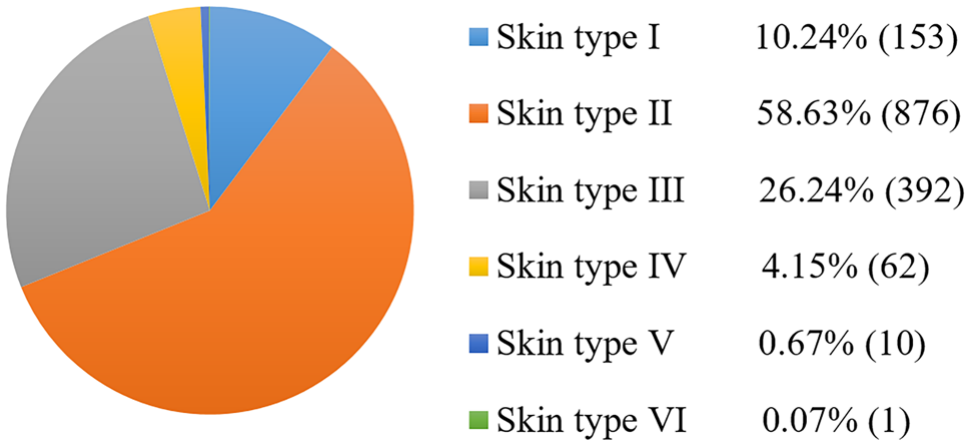
Skin type distribution for 1,494 images in the PAD-UFES-20 dataset [36], according to dermatologist-assigned Fitzpatrick scale labels

**Fig. 7:**
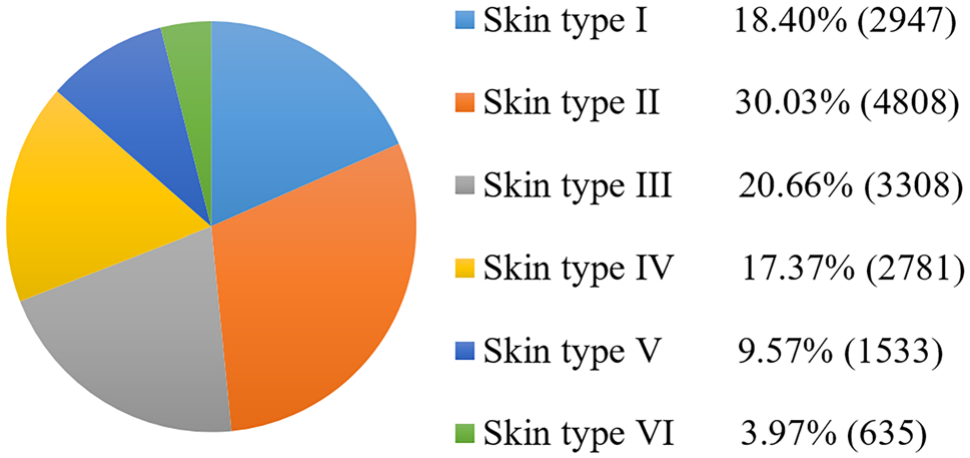
Skin type distribution for 16,012 images in the Fitzpatrick 17 k dataset [26], according to dermatologist-assigned Fitzpatrick scale labels. The original number of images was 16,577, but 565 images had unknown Fitzpatrick skin types

Although the PAD-UFES-20 and Fitzpatrick 17 k datasets provide skin type metadata, they contain far fewer images of darker skin types (e.g. only 635 out of 16,577 images in Fitzpatrick 17 k are of skin type VI, and only one image of skin type VI in PAD-UFES-20). Thus, apart from the lack of reporting of skin type metadata, even if datasets cover skin type information, there is no guarantee that they have enough representation for darker-skinned groups. Figure 6 and Fig. 7 show the distributions of skin types in the PAD-UFES-20 and Fitzpatrick 17 k datasets respectively. It can be seen that skin type VI accounts for the lowest percentage in both datasets: 0.07% in PAD-UFES-20 and 3.97% in Fitzpatrick 17 k. Note that in the Fitzpatrick 17k dataset, the full number of images is 16,577, but 565 images were excluded because they had unknown Fitzpatrick skin types (labeled “-1”).

**Fig. 8:**
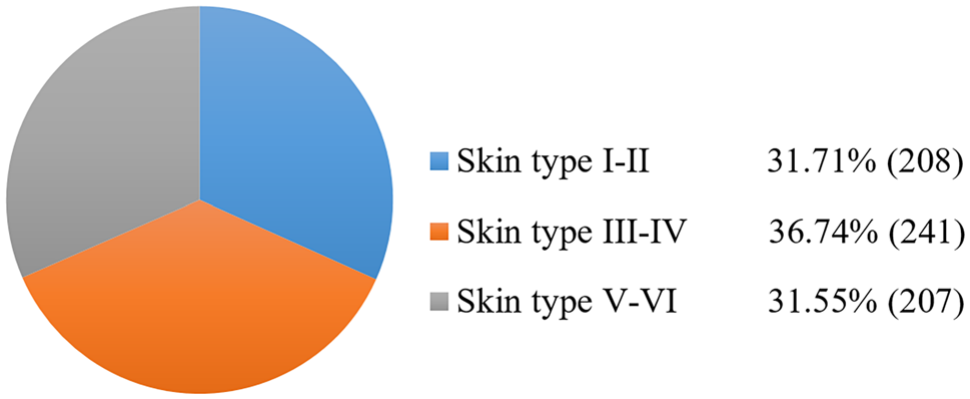
Skin type distribution for the 656 images in the DDI dataset [35], according to dermatologist-assigned Fitzpatrick scale labels

As shown in Fig. 8, the DDI dataset metadata classifies images into three skin type groups, rather than providing exact information for each of the six individual Fitzpatrick skin types. Therefore, although the dataset is balanced concerning these three groups, it does not guarantee that each skin type group is balanced. More importantly, due to its small size, it is not suitable for generalizing deep-learning models for all skin types. In the case of ethnicity labels, it should be noted that ethnicity is different from skin type. To a significant degree, shared ethnicity reflects shared ancestry, but people of the same ethnic group can have a wide range of skin types.

## Conclusions

### Summary of findings

This study is the first review to date that investigates publicly available skin lesion datasets and their metadata in detail for the important issue of inadequate skin type diversity. As these datasets are used for training deep learning models, inadequate skin type diversity within the datasets could affect the performance of the models, in terms of having low accuracy against specific groups of people [35, 97]. To overcome this issue, it is important that, firstly, information about skin type distribution be provided for datasets, and secondly, that skin type diversity be evaluated in detail to facilitate downstream research and ensure that balanced methods are specified for achieving diverse representation before using the datasets for training models.

The issue of inadequate skin type diversity has been discussed in previous works, but without reporting a measurement for each skin type. For example, in the Gender Shades study [28], although the authors used the Fitzpatrick skin type descriptions for their facial image datasets, they just divided the datasets into two skin type groups: darker and lighter. A balanced dataset, FairFace, was created according to different ethnicities, rather than different skin types [29]. Also, the issue of skin lesion datasets was discussed but did not measure skin type diversity for those datasets [1]. Failure to report the distribution of skin types used in a dataset raises concerns about the extent to which different populations are represented in that dataset, and also about the generalizability of machine learning algorithms that have been trained using it.

Our results showed a lack of skin type reporting in all identified skin lesion datasets, except three: PAD-UFES-20, Fitzpatrick 17k, and DDI. Of the skin lesion datasets used in the papers identified in our review, these three are the only ones that provide information about skin type using the Fitzpatrick scale. The shortage of skin lesion datasets including skin type information compared to the large number of skin lesion datasets without it, raises concerns about the high potential for underperformance in AI models trained on these datasets.

However, as shown in the results, two datasets—PAD-UFES-20 and Fitzpatrick 17k—have considerably less representation of darker skin. The DDI dataset reports skin tone distribution in three aggregate groups, rather than for each of the six Fitzpatrick skin types; therefore, exact information about the number of images belonging to each skin type is unavailable. Furthermore, it is too small for training a generalized model that works for all skin types. Nevertheless, none of these three datasets includes information about the ethnicity corresponding to the skin type of each image. Also, the results showed that the distinction between ethnicity and skin type should be restated as one ethnicity can include different skin types.

Deep learning-based models should be developed with fairness and equity in mind, aiming to include a representative distribution of all skin tones. If achieving this balance is not possible, the limitations should be transparently reported, including details on metadata, the training process, and any associated challenges, to ensure clarity regarding the model's performance across different skin tones [97]. This review has shown that skin type diversity in skin lesion image datasets is either unquantifiable (due to lack of skin type metadata in the vast majority of datasets) or inadequate (in the three datasets where metadata is provided). To facilitate the evaluation of skin type diversity, datasets should ideally include dermatologist-assigned Fitzpatrick skin type labels. Compared to classifications like ethnicity and race, Fitzpatrick skin type is relatively clearly defined and provides a more objective basis for establishing diversity.

### Addressing Under-representation

Some metrics such as ISSintra [96] or alternative metrics [97] can be used to measure the skin type diversity of the datasets used for training the AI models. One of the widely used methods in previous works to measure the representation of different skin types in datasets and lack of diversity involves the use of automatic skin type classification methods, such as individual typology angle (ITA). ITA values show an inverse correlation with skin pigmentation and enable the classification of skin color into six groups, ranging from very light to dark skin [98–100].

Finally, to achieve AI models with a fair performance for each skin type, there are methods, including augmentation [101, 102] or adversarial de-biasing [103–105], and balancing datasets [5] to enhance the fairness of the models and create balanced datasets. For example, in addressing dataset imbalance, authors balance minority classes in skin disease datasets through the utilization of class weighting as a data balancing technique [106]. In [5], the authors addressed class imbalance in the clinical dataset using two resampling methods: SMOTE and under-sampling. SMOTE generates synthetic minority examples based on k-nearest neighbors while under-sampling reduces the majority class size to balance the dataset. In the study by Islam et al. [107] normalization, data reduction, and data augmentation are used in pre-processing steps to classify skin lesions from the HAM10000 dataset. In another study, data up-sampling and augmentation methods were used in skin lesion classification using a convolutional neural network (CNN) to improve the classifier's efficiency for the HAM 10000 dataset [108].

To bridge the gap in the under-representation of darker skin tones, [109] used augmentation methods like flipping, cropping, and rotating on two clinical image skin lesion datasets (DermNet NZ and ISIC 2018). This approach increased the inclusion of dark skin tones, resulting in a higher accuracy of 94% for malignancy detection with the augmented datasets. Mohamed et al. [110] showed how balancing the dataset affected skin lesion classification results using two models, MobileNet and DenseNet121, on the HAM10000 dataset. After applying augmentation methods like zooming, rotation, and flipping, the accuracy improved by 20% for DenseNet121 and 10% for MobileNet. Rezk et al. [97] addressed the shortage of dark skin images in dermatology datasets (DermNet NZ, ISIC, Dermatology Atlas) by creating realistic images of darker skin for better diagnosis of skin lesions in people of color. They used style transfer (ST) and deep blending (DB), with ST transferring styles between images and DB blending features from multiple images. Their findings showed that diverse skin color images improved the model's ability to recognize skin tone variations, though geometric transformations alone weren't sufficient to account for all deviations in skin tone distribution in the test set. Rezk et al. [109] used deep learning to generate darker skin tone images from ISIC and DermNet NZ datasets to improve skin cancer detection models. Their results showed that models trained on diverse datasets, including these generated images, provided more accurate diagnoses for people of color. Additionally, other studies have highlighted the benefits of augmentation techniques in balancing datasets and improving diagnostic accuracy [111–114].

### Implications for Future Research

In conclusion, this study underscores the need for sufficient representation of all skin types within datasets, emphasizing the importance of accurate skin type labeling. Achieving fair representation is important for mitigating the underperformance of AI models’ performance, particularly concerning darker skin tones. The disparities in model performance across different skin types can lead to inaccuracies, which may adversely affect the diagnostic accuracy and usability of these models in real-world applications. Strategies such as expanding data collection efforts to ensure adequate representation of diverse skin tones, data augmentation to artificially increase the representation of under-represented skin tones, and transparent reporting to clearly convey the diversity of represented skin tones in datasets, could be employed to achieve more balanced datasets.

### Supplementary Information

Below is the link to the electronic supplementary material.

Supplementary file1 (PDF 202 KB)

## Data Availability

No datasets were generated or analysed during the current study.

## Footnotes

1 The full list of 132 publications is available here: https://arrow.tudublin.ie/engschelecon/15/
