# Supplementary material for: Skin Type Diversity in Skin Lesion Datasets: A Review
Source: Curr Dermatol Rep. 2024 Aug 14;13(3):198–210. doi: 10.1007/s13671-024-00440-0 (PMC11343783; doi:10.1007/s13671-024-00440-0)
Supplement: Supplementary file 1 — Supplementary file1 (PDF 202 KB) [file 13671_2024_440_MOESM1_ESM.pdf]

Technological University Dublin
ARROW@TU Dublin

Other resources School of Electrical and Electronic Engineering

2024-06-29

# Title: List of 132 papers citing one or more skin lesion image datasets

Neda Alipour

Ted Butke

Jane Courtney

Follow this and additional works at: https://arrow.tudublin.ie/engschelecon

Part of the Biomedical Devices and Instrumentation Commons

This work is licensed under a Creative Commons Attribution-NonCommercial-Share Alike 4.0 International License.
Funder: Science Foundation Ireland

**Title: List of 132 papers citing one or more skin lesion image datasets**

Author: Neda Alipour, School of Electrical and Electronic Engineering, Technological University Dublin

Date: 28 June 2024

This document is provided as a supplementary digital resource to accompany the article *Skin Type Diversity in Skin Lesion Datasets: A Review*. The following list contains 132 papers that were identified during a systematic review of papers that use one or more skin lesion image datasets. A partial list of these papers appears in Table 1 of the aforementioned article. The full list is provided here for reference.

Abbas Q, Ramzan F, Ghani MU (2021) Acral melanoma detection using dermoscopic images and convolutional neural networks. Visual Computing for Industry, Biomedicine, and Art, 4, pp.1-12

Abhishek K, Kawahara J, Hamarneh G (2021) Predicting the clinical management of skin lesions using deep learning. Scientific Reports (2021) 11:7769

Ahammed M, Al Mamun M, Uddin MS (2022) A machine learning approach for skin disease detection and classification using image segmentation. Healthcare Analytics, 2, 100122

Ahmad B, Jun S, Palade V, You Q, Mao L, Zhongjie M (2021) Improving skin cancer classification using heavytailed Student t-distribution in generative adversarial networks (TED-GAN). Diagnostics 11112147

Akram T, Lodhi HMJ, Naqvi SR, Naeem S, Alhaisoni M, Ali M, Haider SA, Qadri NN (2020) A multilevel features selection framework for skin lesion classification. Human-centric Computing and Information Sciences (2020) 10:12

Albahar MA (2019) Skin lesion classification using convolutional neural network with novel regularizer. IEEE Access, 7, pp.38306-38313

Aldwgeri A, Abubacker NF (2019) Ensemble of deep convolutional neural network for skin lesion classification in dermoscopy images. In Advances in Visual Informatics: 6th International Visual Informatics Conference, IVIC 2019, Bangi, Malaysia, November 19–21, 2019, Proceedings 6 (pp.214-226). Springer International Publishing

Alenezi F, Armghan A, Polat K (2023) A multi-stage melanoma recognition framework with deep residual neural network and hyperparameter optimization-based decision support in dermoscopy images. Expert Systems with Applications 215 (2023) 119352

Ali SN, Ahmed MT, Paul J, Jahan T, Sani SM, Noor N, Hasan T (2022) Monkeypox skin lesion detection using deep learning models: A feasibility study. arXiv preprint arXiv:2207.03342

Andrade C, Teixeira LF, Vasconcelos MJM, Rosado L (2020) Data augmentation using adversarial image-toimage translation for the segmentation of mobile-acquired dermatological images. Journal of Imaging, 7(1), p.2

Ashim LK, Suresh N, Prasannakumar CV (2021) A comparative analysis of various transfer learning approaches skin cancer detection. In 2021 5th International Conference on Trends in Electronics and Informatics (ICOEI) (pp. 1379-1385). IEEE

Ashraf R, Afzal S, Rehman AU, Gul S, Baber J, Bakhtyar M, Mehmood I, Song OY, Maqsood M (2020) Regionof-interest based transfer learning assisted framework for skin cancer detection. IEEE Access, 8, pp.147858147871

Bagheri F, Tarokh MJ, Ziaratban M (2021) Skin lesion segmentation based on mask RCNN, Multi Atrous FullCNN, and a geodesic method. International Journal of Imaging Systems and Technology, 31(3), pp.1609-1624

Banerjee S, Singh SK, Chakraborty A, Das A, Bag R (2020) Melanoma diagnosis using deep learning and fuzzy logic. Diagnostics 2020, 10, 577

Barata C, Celebi ME, Marques JS (2014) Improving dermoscopy image classification using color constancy. IEEE Journal of Biomedical and Health Informatics, 19(3), pp.1146-1152

Barata C, Celebi ME, Marques JS (2021) Explainable skin lesion diagnosis using taxonomies. Pattern Recognition 110 (2021)107413

Begum M, Dhivya A, Krishnan AJ, Keerthana SD (2021) Automated detection of skin and nail disorders using convolutional neural networks. In 2021 5th International Conference on Trends in Electronics and Informatics (ICOEI) (pp. 1309-1316). IEEE

Behara K, Bhero E, Agee JT (2024) An Improved Skin Lesion Classification Using a Hybrid Approach with Active Contour Snake Model and Lightweight Attention-Guided Capsule Networks. Diagnostics. 14(6):636.

Bisla D, Choromanska A, Berman RS, Stein JA, Polsky D (2019) Towards automated melanoma detection with deep learning: Data purification and augmentation. In Proceedings of the IEEE/CVF conference on computer vision and pattern recognition workshops

Bissoto A, Valle E, Avila S (2020) Debiasing skin lesion datasets and models? Not so fast. In Proceedings of the IEEE/CVF Conference on Computer Vision and Pattern Recognition Workshops (pp.740-741)

Belouadah E, Popescu A, Aggarwal U, Saci L (2020) Active class incremental learning for imbalanced datasets. In European Conference on Computer Vision (pp. 146-162). Cham: Springer International Publishing.

Brinker TJ, Hekler A, Enk AH, Klode J, Hauschild A, Berking C, Schilling B, Haferkamp S, Schadendorf D, Fröhling S, Utikal JS (2019) A convolutional neural network trained with dermoscopic images performed on par with 145 dermatologists in a clinical melanoma image classification task. European Journal of Cancer, 111, pp.148-154

Brinker TJ, Hekler A, Enk AH, Klode J, Hauschild A, Berking C, Schilling B, Haferkamp S, Schadendorf D, Holland-Letz T, Utikal JS (2019) Deep learning outperformed 136 of 157 dermatologists in a head-to-head dermoscopic melanoma image classification task. European Journal of Cancer, 113, pp.47-54

Brinker TJ, Hekler A, Hauschild A, Berking C, Schilling B, Enk AH, Haferkamp S, Karoglan A, von Kalle C, Weichenthal M, Sattler E (2019) Comparing artificial intelligence algorithms to 157 German dermatologists: the melanoma classification benchmark. European Journal of Cancer, 111, pp.30-37

Cano E, Mendoza-Avilés J, Areiza M, Guerra N, Mendoza-Valdés JL, Rovetto CA (2021) Multi skin lesions classification using fine-tuning and data-augmentation applying NASNet. PeerJ Comput. Sci. 7:e371

Chabi Adjobo E, Sanda Mahama AT, Gouton P, Tossa J (2022) Towards accurate skin lesion classification across all skin categories using a PCNN fusion-based data augmentation approach. Computers 2022, 11, 44

Chaturvedi SS, Gupta K, Prasad PS (2021) Skin lesion analyser: an efficient seven-way multi-class skin cancer classification using MobileNet. In Advanced Machine Learning Technologies and Applications: Proceedings of AMLTA 2020 (pp. 165-176). Springer Singapore.

Chiu CH, Chen YJ, Wu Y, Shi Y, Ho TY (2024) Achieve fairness without demographics for dermatological disease diagnosis. Medical Image Analysis. 95:103188.

Codella NC, Gutman D, Celebi ME, Helba B, Marchetti MA, Dusza SW, Kalloo A, Liopyris K, Mishra N, Kittler H, Halpern A (2018) April. Skin lesion analysis toward melanoma detection: A challenge at the 2017 international symposium on biomedical imaging (ISBI), hosted by the international skin imaging collaboration (ISIC). In 2018 IEEE 15th international symposium on biomedical imaging (ISBI 2018) (pp.168-172). IEEE

Combalia M, Codella NC, Rotemberg V, Helba B, Vilaplana V, Reiter O, Carrera C, Barreiro A, Halpern AC, Puig S, Malvehy J (2019) BCN20000: Dermoscopic lesions in the wild. arXiv preprint arXiv:1908.02288

Coronado-Gutiérrez D, López C, Burgos-Artizzu XP (2021) Skin cancer high-risk patient screening from dermoscopic images via Artificial Intelligence: An online study. medRxiv, 2021-02

Courtenay LA, Barbero-García I, Aramendi J, González-Aguilera D, Rodríguez-Martín M, Rodríguez-Gonzalvez P, Cañueto J, Román-Curto C (2022) A Novel Approach for the Shape Characterisation of Non-Melanoma Skin Lesions Using Elliptic Fourier Analyses and Clinical Images. Journal of Clinical Medicine 2022, 11, 4392 de Faria SM, Filipe JN, Pereira PM, Tavora LM, Assuncao PA, Santos MO, Fonseca-Pinto R, Santiago F, Dominguez V, Henrique M (2019) Light field image dataset of skin lesions. In 2019 41st Annual International Conference of the IEEE Engineering in Medicine and Biology Society (EMBC) (pp.3905-3908). IEEE

Daneshjou R, Vodrahalli K, Novoa RA, Jenkins M, Liang W, Rotemberg V, Ko J, Swetter SM, Bailey EE, Gevaert O, Mukherjee P (2022) Disparities in dermatology AI performance on a diverse, curated clinical image set. Science advances. 8(31):eabq6147.

Ding J, Song J, Li J, Tang J, Guo F (2022) Two-stage deep neural network via ensemble learning for melanoma classification. Frontiers in Bioengineering and Biotechnology, 9, 758495

El Gannour O, Hamida S, Lamalem Y, Mahjoubi MA, Cherradi B, Raihani A (2024) Improving skin diseases prediction through data balancing via classes weighting and transfer learning. Bulletin of Electrical Engineering and Informatics 13(1):628-37.

Fan X, Dai M, Liu C, Wu F, Yan X, Feng Y, Feng Y, Su B (2019) Effect of image noise on the classification of skin lesions using deep convolutional neural networks. Tsinghua Science and Technology, 25(3), pp.425-434

Fisher RB, Rees J, Bertrand A (2020) Classification of ten skin lesion classes: Hierarchical KNN versus deep net. In Medical Image Understanding and Analysis*:* 23rd Conference, MIUA 2019, Liverpool, UK, July 24–26, 2019, Proceedings 23 (pp.86-98). Springer International Publishing

Fogelberg K, Chamarthi S, Maron RC, Niebling J, Brinker TJ (2023) Domain shifts in dermoscopic skin cancer datasets: Evaluation of essential limitations for clinical translation. New Biotechnology, 76, pp.106-117

França LDR (2018) Neuroevolution of Augmenting Topologies Applied to the Detection of Cancer in Medical Images. Dissertation, Universidade Federal Rural de Pernambuco – UFRPE

Ge Z, Demyanov S, Bozorgtabar B, Abedini M, Chakravorty R, Bowling A, Garnavi R (2017) Exploiting local and generic features for accurate skin lesions classification using clinical and dermoscopy imaging. In 2017 IEEE 14th International Symposium on Biomedical Imaging (ISBI 2017) (pp.986-990). IEEE

Giotis I, Molders N, Land S, Biehl M, Jonkman MF, Petkov N (2015) MED-NODE: A computer-assisted melanoma diagnosis system using non-dermoscopic images. Expert Systems with Applications, 42(19), pp.65786585

Gonzalez-Diaz I (2018) Dermaknet: Incorporating the knowledge of dermatologists to convolutional neural networks for skin lesion diagnosis. IEEE Journal of Biomedical and Health Informatics, 23(2), pp.547-559

Goyal M, Knackstedt T, Yan S, Hassanpour S (2020) Artificial intelligence-based image classification methods for diagnosis of skin cancer: Challenges and opportunities. Computers in Biology and Medicine 127 (2020) 104065

Goyal M, Yap MH, Hassanpour S (2018) Deep Learning Methods and Applications for Region of Interest Detection in Dermoscopic Images. arXiv preprint arXiv:1807.10711

Groh M, Harris C, Soenksen L, Lau F, Han R, Kim A, Koochek A, Badri O (2021) Evaluating deep neural networks trained on clinical images in dermatology with the Fitzpatrick 17k dataset. In Proceedings of the IEEE/CVF Conference on Computer Vision and Pattern Recognition (pp.1820-1828)

Gu Y, Ge Z, Bonnington CP, Zhou J (2019) Progressive transfer learning and adversarial domain adaptation for cross-domain skin disease classification. IEEE Journal of Biomedical and Health Informatics, 24(5), pp.13791393

Gu Y, Zhou J, Qian B (2017) Melanoma detection based on mahalanobis distance learning and constrained graph regularized nonnegative matrix factorization. In 2017 IEEE Winter Conference on Applications of Computer Vision (WACV) (pp.797-805). IEEE

Gutman D, Codella NC, Celebi E, Helba B, Marchetti M, Mishra N, Halpern A (2016) Skin lesion analysis toward melanoma detection: A challenge at the International Symposium on Biomedical Imaging (ISBI) 2016, hosted by the International Skin Imaging Collaboration (ISIC). arXiv preprint arXiv:1605.01397

Han SS, Kim MS, Lim W, Park GH, Park I, Chang SE (2018) Classification of the clinical images for benign and malignant cutaneous tumors using a deep learning algorithm. Journal of Investigative Dermatology, 138(7), pp.1529-1538

Han SS, Park GH, Lim W, Kim MS, Na JI, Park I, Chang SE (2018) Deep neural networks show an equivalent and often superior performance to dermatologists in onychomycosis diagnosis: Automatic construction of onychomycosis datasets by region-based convolutional deep neural network. PLOS ONE 13(1): e0191493

Han SS, Park I, Chang SE, Lim W, Kim MS, Park GH, Chae JB, Huh CH, Na JI (2020) Augmented intelligence dermatology: deep neural networks empower medical professionals in diagnosing skin cancer and predicting treatment options for 134 skin disorders. Journal of Investigative Dermatology, 140(9), pp.1753-1761

Harangi B (2018) Skin lesion classification with ensembles of deep convolutional neural networks. Journal of Biomedical Informatics, 86, pp.25-32

Hasan MK, Elahi MTE, Alam MA, Jawad MT, Martí R (2022) DermoExpert: Skin lesion classification using a hybrid convolutional neural network through segmentation, transfer learning, and augmentation. Informatics in Medicine Unlocked 28 (2022) 100819

Hasan MR, Fatemi MI, Monirujjaman Khan M, Kaur M, Zaguia A (2021) Comparative analysis of skin cancer (benign vs. malignant) detection using convolutional neural networks. Journal of Healthcare Engineering, 2021

He X, Wang S, Shi S, Tang Z, Wang Y, Zhao Z, Dai J, Ni R, Zhang X, Liu X, Wu Z (2019) Computer-aided clinical skin disease diagnosis using CNN and object detection models. In 2019 IEEE International Conference on Big Data (pp.4839-4844). IEEE

Hilmy MA, Sasongko PS (2019) Ensembles of convolutional neural networks for skin lesion dermoscopy images classification. In 2019 3rd International Conference on Informatics and Computational Sciences (ICICoS) (pp.16). IEEE

Hosny KM, Kassem MA, Foaud MM (2019) Classification of skin lesions using transfer learning and augmentation with Alex-net. PLOS ONE 14(5): e0217293

Hosny KM, Kassem MA, Foaud MM (2020) Skin melanoma classification using ROI and data augmentation with deep convolutional neural networks. Multimedia Tools and Applications, 79, pp.24029-24055

Islam MK, Ali MS, Ali MM, Haque MF, Das AA, Hossain MM, Duranta DS, Rahman MA (2021) Melanoma skin lesions classification using deep convolutional neural network with transfer learning. In 2021 1st International Conference on Artificial Intelligence and Data Analytics (CAIDA). pp. 48-53. IEEE.

Jiang F, Zhou F, Qin J, Wang T, Lei B (2019) Decision-augmented generative adversarial network for skin lesion segmentation. In 2019 IEEE 16th International Symposium on Biomedical Imaging (ISBI 2019) (pp.447-450). IEEE

Jiang Y, Huang R, Shi J (2021) EfficientNet-based model with test time augmentation for cancer detection. In 2021 IEEE 2nd International Conference on Big Data, Artificial Intelligence and Internet of Things Engineering (ICBAIE) (pp.548-551). IEEE

Kawahara J, BenTaieb A, Hamarneh G (2016) Deep features to classify skin lesions. In 2016 IEEE 13th International Symposium on Biomedical Imaging (ISBI) (pp.1397-1400). IEEE

Kawahara J, Daneshvar S, Argenziano G, Hamarneh G (2018) Seven-point checklist and skin lesion classification using multitask multimodal neural nets. IEEE Journal of Biomedical and Health Informatics, 23(2), pp.538-546

Khan MA, Akram T, Sharif M, Saba T, Javed K, Lali IU, Tanik UJ, Rehman A (2019) Construction of saliency map and hybrid set of features for efficient segmentation and classification of skin lesion. Microscopy Research and Technique, 82(6), pp.741-763

Khan MA, Akram T, Sharif M, Shahzad A, Aurangzeb K, Alhussein M, Haider SI, Altamrah A (2018) An implementation of normal distribution based segmentation and entropy controlled features selection for skin lesion detection and classification. BMC Cancer, 18, pp.1-20

Khan MA, Sharif M, Akram T, Damaševičius R, Maskeliūnas R (2021) Skin lesion segmentation and multiclass classification using deep learning features and improved moth flame optimization. Diagnostics, 11(5), p.811

Krohling B, Castro PB, Pacheco AG, Krohling RA (2021) A smartphone based application for skin cancer classification using deep learning with clinical images and lesion information. arXiv preprint arXiv:2104.14353

Lézoray O, Revenu M, Desvignes M (2014) Graph-based skin lesion segmentation of multispectral dermoscopic images. In 2014 IEEE International Conference on Image Processing (ICIP) (pp.897-901). IEEE

Li H, Pan Y, Zhao J, Zhang L (2021) Skin disease diagnosis with deep learning: A review. Neurocomputing, 464, pp.364-393

Liao H (2016) A deep learning approach to universal skin disease classification. University of Rochester Department of Computer Science, CSC

Liao H, Li Y, Luo J (2016) Skin disease classification versus skin lesion characterization: Achieving robust diagnosis using multi-label deep neural networks. In 2016 23rd International Conference on Pattern Recognition (ICPR) (pp.355-360). IEEE

Liu Y, Primiero CA, Kulkarni V, Soyer HP, Betz-Stablein B (2023) Artificial intelligence for the classification of pigmented skin lesions in populations with skin of color: a systematic review. Dermatology. 239(4):499-513.

Lopez AR, Giro-i-Nieto X, Burdick J, Marques O (2017) Skin lesion classification from dermoscopic images using deep learning techniques. In 2017 13th IASTED International Conference on Biomedical Engineering (BioMed) (pp.49-54). IEEE

Lucius M, De All J, De All JA, Belvisi M, Radizza L, Lanfranconi M, Lorenzatti , Galmarini CM (2020) Deep neural frameworks improve the accuracy of general practitioners in the classification of pigmented skin lesions. Diagnostics 10110969

Luo W, Yang M (2018) Fast skin lesion segmentation via fully convolutional network with residual architecture and CRF. In 2018 24th International Conference on Pattern Recognition (ICPR) (pp.1438-1443). IEEE

Mahajan K, Sharma M, Vig L (2020) Meta-dermdiagnosis: Few-shot skin disease identification using metalearning. In Proceedings of the IEEE/CVF Conference on Computer Vision and Pattern Recognition Workshops (pp.730-731)

Mahum R, Aladhadh S (2022) Skin lesion detection using hand-crafted and DL-based features fusion and LSTM. Diagnostics 12122974

Maiti R, Agarwal P, Kumar RR, Bhat A (2021) Detection of skin cancer using neural architecture search with model quantization. In 2021 5th International Conference on Intelligent Computing and Control Systems (ICICCS) (pp.1807-1814). IEEE

Maron RC, Haggenmüller S, von Kalle C, Utikal JS, Meier F, Gellrich FF, Hauschild A, French LE, Schlaak M, Ghoreschi K, Kutzner H (2021) Robustness of convolutional neural networks in recognition of pigmented skin lesions. European Journal of Cancer, 145, pp.81-91

Mazoure B, Mazoure A, Bédard J, Makarenkov V (2022) DUNEScan: a web server for uncertainty estimation in skin cancer detection with deep neural networks. Scientific Reports, 12(1), p.179

Mendes DB, da Silva NC (2018) Skin lesions classification using convolutional neural networks in clinical images. arXiv preprint arXiv:1812.02316

Mendonça T, Ferreira PM, Marques JS, Marcal AR, Rozeira J (2013) PH 2-A dermoscopic image database for research and benchmarking. In 2013 35th Annual International Conference of the IEEE Engineering in Medicine and Biology Society (EMBC) (pp.5437-5440). IEEE

Mikołajczyk A, Grochowski M (2018) Data augmentation for improving deep learning in image classification problem. In 2018 International Interdisciplinary PhD Workshop (IIPhDW) (pp.117-122). IEEE

Milantev S, Olyunin V, Bykov I, Milanteva N, Bessmertny I (2020) Skin lesion analysis using ensemble of CNN with dermoscopic images and metadata. Proceedings of the 12th Majorov International Conference on Software Engineering and Computer Systems, December 10–11, 2020, Online Saint Petersburg, Russia

Milczarski P, Beczkowski M, Borowski N (2021) Enhancing dermoscopic features classification in images using invariant dataset augmentation and convolutional neural networks. In International Conference on Neural Information Processing (pp.403-417). Springer International Publishing

Moataz L, Salama GI, Abd Elazeem MH (2021) Skin cancer diseases classification using deep convolutional neural network with transfer learning model. In Journal of Physics: Conference Series (Vol. 2128, No. 1, p. 012013). IOP Publishing

Mohammed SS, Al-Tuwaijari JM (2021) Skin disease classification system based on machine learning technique: A survey. In IOP Conference Series: Materials Science and Engineering (Vol. 1076, No. 1, p.012045). IOP Publishing

Morales-Forero AN, Mayorga JH, Rueda LJ, Sanchez-zapata MJ, Arboleda AJ, Bassetto S, Coatanea E (2023) Exposing Racial disparities of AI-based imaging classifiers for pigmented skin lesions diagnosis.

Nedelcu T, Vasconcelos M, Carreiro A (2020) Multi-dataset training for skin lesion classification on multimodal and multitask deep learning. In Proceedings of the 6th World Congress on Electrical Engineering and Computer Systems and Sciences (EECSS'20), Prague, Czech Republic (pp.13-15)

Nunnari F, Sonntag D (2019) A CNN toolbox for skin cancer classification. arXiv preprint arXiv:1908.08187

Pacheco AG, Ali AR, Trappenberg T (2019) Skin cancer detection based on deep learning and entropy to detect outlier samples. arXiv preprint arXiv:1909.04525

Pacheco AG, Lima GR, Salomao AS, Krohling B, Biral IP, de Angelo GG, Alves Jr FC, Esgario JG, Simora AC, Castro PB, Rodrigues FB (2020) PAD-UFES-20: A skin lesion dataset composed of patient data and clinical images collected from smartphones. Data in Brief 32 (2020) 106221

Perez E, Reyes O, Ventura S (2021) Convolutional neural networks for the automatic diagnosis of melanoma: An extensive experimental study. Medical Image Analysis 67 (2021) 101858

Pham TC, Luong CM, Visani M, Hoang VD (2018) Deep CNN and data augmentation for skin lesion classification. In Intelligent Information and Database Systems: 10th Asian Conference, ACIIDS 2018, Dong Hoi City, Vietnam, March 19-21, 2018, Proceedings, Part II 10 (pp.573-582). Springer International Publishing

Pour MP, Seker H (2020) Transform domain representation-driven convolutional neural networks for skin lesion segmentation. Expert Systems with Applications 144 (2020) 113129

Rahman Z, Hossain MS, Islam MR, Hasan MM, Hridhee RA (2021) An approach for multiclass skin lesion classification based on ensemble learning. Informatics in Medicine Unlocked 25 (2021) 100659

Ren Z, Guo Y, Stella XY, Whitney D (2021) Improve image-based skin cancer diagnosis with generative selfsupervised learning. In 2021 IEEE/ACM Conference on Connected Health: Applications, Systems and Engineering Technologies (CHASE) (pp.23-34). IEEE

Rezk E, Eltorki M, El-Dakhakhni W (2022) Improving skin color diversity in cancer detection: deep learning approach. JMIR Dermatology. 5(3):e39143.

Rezk E, Eltorki M, El-Dakhakhni W (2022) Leveraging artificial intelligence to improve the diversity of dermatological skin color pathology: protocol for an algorithm development and validation study. JMIR Research Protocols. 11(3):e34896.

Rotemberg V, Kurtansky N, Betz-Stablein B, Caffery L, Chousakos E, Codella N, Combalia M, Dusza S, Guitera P, Gutman D, Halpern A (2021) A patient-centric dataset of images and metadata for identifying melanomas using clinical context. Scientific Data (2021) 8:34

Sae-Lim W, Wettayaprasit W, Aiyarak P (2019) Convolutional neural networks using MobileNet for skin lesion classification. In 2019 16th International Joint Conference on Computer Science and Software Engineering (JCSSE) (pp.242-247). IEEE

Saez A, Serrano C, Acha B (2014) Model-based classification methods of global patterns in dermoscopic images. IEEE Transactions on Medical Imaging, 33(5), pp.1137-1147

Saha A, Prasad P, Thabit A (2020) Leveraging adaptive color augmentation in convolutional neural networks for deep skin lesion segmentation. In 2020 IEEE 17th International Symposium on Biomedical Imaging (ISBI) (pp.2014-2017). IEEE

Sarker MMK, Rashwan HA, Akram F, Singh VK, Banu SF, Chowdhury FU, Choudhury KA, Chambon S, Radeva P, Puig D, Abdel-Nasser M (2021) SLSNet: Skin lesion segmentation using a lightweight generative adversarial network. Expert Systems with Applications 183 (2021) 115433

Sayed GI, Soliman MM, Hassanien AE (2021) A novel melanoma prediction model for imbalanced data using optimized SqueezeNet by bald eagle search optimization. Computers in Biology and Medicine 136 (2021) 104712

Shah V, Autee P, Sonawane P (2020) Detection of melanoma from skin lesion images using deep learning techniques. In 2020 International Conference on Data Science and Engineering (ICDSE) (pp.1-8). IEEE

Shoieb DA, Youssef SM (2018) An enhanced hybrid model for skin diagnosis using deep convolution neural network. In 2018 9th Cairo International Biomedical Engineering Conference (CIBEC) (pp.37-40). IEEE

Shorfuzzaman M (2022) An explainable stacked ensemble of deep learning models for improved melanoma skin cancer detection. Multimedia Systems, 28(4), pp.1309-1323

Steppan J, Hanke S (2021) Analysis of skin lesion images with deep learning. arXiv preprint arXiv:2101.03814

Sun Q, Huang C, Chen M, Xu H, Yang Y (2021) Skin lesion classification using additional patient information. BioMed Research International

Sun X, Yang J, Sun M, Wang K (2016) A benchmark for automatic visual classification of clinical skin disease images. In Computer Vision–ECCV 2016: 14th European Conference, Amsterdam, The Netherlands, October 1114, 2016, Proceedings, Part VI 14 (pp.206-222). Springer International Publishing

Tan TY, Zhang L, Lim CP, Fielding B, Yu Y, Anderson E (2019) Evolving ensemble models for image segmentation using enhanced particle swarm optimization. IEEE Access, *7*, pp.34004-34019

Taylor SC (2002) Skin of color: biology, structure, function, and implications for dermatologic disease. Journal of the American Academy of Dermatology, 46(2), pp.S41-S62.

Tschandl P, Rosendahl C, Kittler H (2018) The HAM10000 dataset, a large collection of multi-source dermatoscopic images of common pigmented skin lesions. Scientific Data, 5(1), pp.1-9

Ünver HM, Ayan E (2019) Skin lesion segmentation in dermoscopic images with combination of YOLO and grabcut algorithm. Diagnostics 9030072

Valle E, Fornaciali M, Menegola A, Tavares J, Bittencourt FV, Li LT, Avila S (2020) Data, depth, and design: Learning reliable models for skin lesion analysis. Neurocomputing, 383, pp.303-313

Vasconcelos MJM, Rosado L, Ferreira M (2014) Principal axes-based asymmetry assessment methodology for skin lesion image analysis. In International Symposium on Visual Computing (pp.21-31). Springer International Publishing

Veronese F, Branciforti F, Zavattaro E, Tarantino V, Romano V, Meiburger KM, Salvi M, Seoni S, Savoia P (2021) The role in teledermoscopy of an inexpensive and easy-to-use smartphone device for the classification of three types of skin lesions using convolutional neural networks. Diagnostics 11030451

Villa-Pulgarin JP, Ruales-Torres AA, Arias-Garzon D, Bravo-Ortiz MA, Arteaga-Arteaga HB, Mora-Rubio A, Alzate-Grisales JA, Mercado-Ruiz E, Hassaballah M, Orozco-Arias S, Cardona-Morales O (2022) Optimized convolutional neural network models for skin lesion classification. Comput. Mater. Contin, 70(2), pp.2131-2148

Waweru AK, Ahmed K, Miao Y, Kawan P (2020) Deep learning in skin lesion analysis towards cancer detection. In 2020 24th International Conference Information Visualisation (IV) (pp.740-745). IEEE

Wu H, Pan J, Li Z, Wen Z, Qin J (2020) Automated skin lesion segmentation via an adaptive dual attention module. IEEE Transactions on Medical Imaging, 40(1), pp.357-370

Xie B, He X, Zhao S, Li Y, Su J, Zhao X, Kuang Y, Wang Y, Chen X (2019) XiangyaDerm: a clinical image dataset of asian race for skin disease aided diagnosis. In Large-Scale Annotation of Biomedical Data and Expert Label Synthesis and Hardware Aware Learning for Medical Imaging and Computer Assisted Intervention: International Workshops, LABELS 2019, HAL-MICCAI 2019, and CuRIOUS 2019, Held in Conjunction with MICCAI 2019, Shenzhen, China, October 13 and 17, 2019, Proceedings 4 (pp.22-31). Springer International Publishing

Yang J, Wu X, Liang J, Sun X, Cheng MM, Rosin PL, Wang L (2019) Self-paced balance learning for clinical skin disease recognition. IEEE Transactions on Neural Networks and Learning Systems, 31(8), pp.2832-2846

Yang Y, Wang J, Xie F, Liu J, Shu C, Wang Y, Zheng Y, Zhang H (2021) A convolutional neural network trained with dermoscopic images of psoriasis performed on par with 230 dermatologists. Computers in Biology and Medicine 139 (2021) 104924

Yao P, Shen S, Xu M, Liu P, Zhang F, Xing J, Shao P, Kaffenberger B, Xu RX (2021) Single model deep learning on imbalanced small datasets for skin lesion classification. IEEE Transactions on Medical Imaging, 41(5), pp.1242-1254

Yoon C, Hamarneh G, Garbi R (2019) Generalizable feature learning in the presence of data bias and domain class imbalance with application to skin lesion classification. In Medical Image Computing and Computer Assisted Intervention–MICCAI 2019: 22nd International Conference, Shenzhen, China, October 13–17, 2019, Proceedings, Part IV 22 (pp.365-373). Springer International Publishing

Yu C, Tang M, Yang S, Wang M, Xu Z, Yan J, Chen H, Yang Y, Zeng XJ, Li X (2021) Towards better dermoscopic image feature representation learning for melanoma classification. In Neural Information Processing: 28th International Conference, ICONIP 2021, Sanur, Bali, Indonesia, December 8–12, 2021, Proceedings, Part IV 28 (pp.550-561). Springer International Publishing

Yu C, Yang S, Kim W, Jung J, Chung KY, Lee SW, Oh B (2018) Acral melanoma detection using a convolutional neural network for dermoscopy images. PLOS ONE 13(3): e0193321

Zhang J, Petitjean C, Ainouz S (2020) Kappa loss for skin lesion segmentation in fully convolutional network. In 2020 IEEE 17th International Symposium on Biomedical Imaging (ISBI) (pp.2001-2004). IEEE

Zhang L, Mishra S, Zhang T, Zhang Y, Zhang D, Lv Y, Lv M, Guan N, Hu XS, Chen DZ, Han X (2021) Design and assessment of convolutional neural network based methods for vitiligo diagnosis. Frontiers in Medicine 754202

Zhao C, Shuai R, Ma L, Liu W, Hu D, Wu M (2021) Dermoscopy image classification based on StyleGAN and DenseNet201. IEEE Access, 9, pp.8
